# Detecting early structural and functional myocardial alterations in patients with repaired tetralogy of fallot: a prospective MRI study

**DOI:** 10.1038/s41598-026-50982-5

**Published:** 2026-05-06

**Authors:** Hannah E. Kappler, Nadja Kocher, Sebastian Berg, Markus Uhl, Marius Menza, Bernd Jung, Christopher L. Schlett, Christoph Zürn, Brigitte Stiller, Charlotte Wintergerst, Jana Taron

**Affiliations:** 1 Department of Congenital Heart Defects and Pediatric Cardiology, Faculty of Medicine, University Heart Center Freiburg, Medical Center-University of Freiburg, University of Freiburg, Freiburg, Germany; 2 Department of Radiology, Kantonsspital Aarau, Aarau, Switzerland; 3 Institut für Bildgebende Diagnostik (IBID), Diakoniekrankenhaus, Freiburg, Germany; 4 Department of Radiology, Pediatric Radiology, and Interventional Radiology, St. Josefskrankenhaus, Freiburg, Germany; 5 Department of Radiology, Medical Physics, Faculty of Medicine, Medical Center-University of Freiburg, University of Freiburg, Freiburg, Germany; 6 Department of Diagnostic, Interventional and Pediatric Radiology, University Hospital Bern, Bern, Switzerland; 7 Department of Diagnostic and Interventional Radiology, Faculty of Medicine, Medical Center-University of Freiburg, University of Freiburg, Freiburg, Germany

**Keywords:** Tetralogy of Fallot, MRI, Cardiac function, Tissue phase mapping, T1 mapping, Fibrosis, Cardiology, Diseases, Medical research

## Abstract

In patients with repaired tetralogy of Fallot (rTOF), right ventricular (RV) fibrosis has been associated with ventricular tachycardia, heart failure, and sudden cardiac death. Here, we aimed to identify links between diffuse myocardial fibrosis and regional mechanical dysfunction by cardiac magnetic resonance imaging (CMR) in rTOF. Clinical CMR was extended in 17 consecutive rTOF patients (median age 20 years, 4 females) to include biventricular native T1 (nT1) mapping as indicator of diffuse fibrosis, and tissue phase mapping (TPM) for quantification of biventricular segmental, directional systolic/diastolic myocardial velocities. Patients demonstrated smaller peak velocities than healthy volunteers in both ventricles (e.g. LV-base circumferential peak-systolic velocity: median [IQR] −0.9 [1.5] vs. −3.0 [1.2] cm/s, *p* < 0.001). RV peak velocities were reduced even in patients with normal RV ejection fraction (e.g. RV-mid long-axis peak-diastolic velocity: −3.3 [1.2] vs. −6.9 [1.8] cm/s, *p* = 0.001). Global LV nT1 was higher in patients versus controls (1029 [48] vs. 993 [28] ms, *p* = 0.013). Within the rTOF group, fibrosis in lateral LV correlated with shorter time-to-peak-velocity (TTP), whereas fibrosis in inferior RV correlated with longer TTP. Therefore, in rTOF TPM facilitates early diagnosis of impaired systolic and diastolic myocardial function while diffuse fibrosis has differential effects on RV and LV mechanics.

**Supplementary Information:**

The online version contains supplementary material available at 10.1038/s41598-026-50982-5.

## Introduction

Tetralogy of Fallot (TOF) is the most common cyanotic congenital heart defect with a prevalence of 0.34 per 1000 live births^1^. With surgical repair performed within the first year of life, survival rates and quality of life have increased significantly in the past decades. However, relevant morbidity for patients with repaired TOF (rTOF) remains due to ventricular tachycardia, (right) heart failure, and sudden cardiac death^2–4^. Patients with rTOF have demonstrated enhanced diffuse myocardial fibrosis in the right ventricle (RV) also in areas remote from surgical scars, presumably due to pulmonary regurgitation secondary to transanular patch surgery and ensuing volume overload^5,6^. In previous studies, this RV fibrosis was indicative of the above named complications with poorer clinical outcome overall in patients with rTOF^7–10^, however the underlying mechanisms are elusive.

Cardiac magnetic resonance imaging (CMR) has emerged as a reliable method for RV assessment in patients with rTOF^11^, and native T1 (nT1) mapping as well as contrast-based calculation of the extracellular volume [ECV] of the myocardium have recently been established as quantitative surrogate parameters for diffuse fibrosis^12–14^. In patients with rTOF, studies have indicated increased nT1/ECV^8,9,13–18^ in the RV, confirming findings of increased myocardial fibrosis in histological assessment of TOF myocardium^5,6,19^. Increased nT1/ECV in the RV and, in some studies, the left ventricle (LV) were repeatedly associated with adverse cardiac events in patients with rTOF^8,9,13–17,20–22^.

Patients with rTOF show impaired ejection fraction (EF) of the RV, and occasionally also of the LV^23^. Histologically apparent fibrosis in the RV outflow tract (RVOT), as well as scar-associated fibrosis measured by late gadolinium enhancement (LGE) was inversely correlated with RV EF^9,10,24,25^ and LV EF^9^, but not with RV global longitudinal strain^24^. In contrast, in two rat models of volume overload, RV (dys-)function was not associated with RV fibrosis^26,27^. It remains unclear, whether the extent of diffuse myocardial fibrosis influences regional myocardial velocities in patients with TOF and whether it may, in parts, be directly responsible for the myocardial dysfunction described after TOF repair.

The aim of this study was, therefore, to identify links between regional systolic and diastolic mechanical function and diffuse myocardial fibrosis in LV and RV of patients with rTOF. Using, amongst others, novel CMR sequences enabling direct measurement of myocardial systolic and diastolic velocities with high spatial and temporal resolution in the RV by tissue phase mapping (TPM)^28^ we (1) assessed myocardial function parameters (EF, segmental systolic/diastolic velocities), (2) evaluated the presence and quantity of myocardial fibrosis, and (3) correlated mechanical function with the extent of fibrosis in patients with rTOF compared to healthy volunteers.

## Patients and methods

### Study design and subjects

This monocentric, prospective study received approval from the Ethics Committee of the Medical Center – University of Freiburg, Germany, and adhered to the Declaration of Helsinki. All participants/legal guardians gave written informed consent before participating in this study.

We enrolled 17 consecutive patients with rTOF requiring clinically indicated CMR between June 2020 and September 2022. Clinical indication for CMR in all patients was assessment of RV size and function, pulmonary valve function, as well as RVOT and pulmonary artery morphology due to impaired imaging quality and/or progressive RV dilation in transthoracic echocardiography. Exclusion criteria included lacking written informed consent, additional significant cardiac malformations, as well as known contraindications to MRI or to gadolinium-containing contrast agents.

For the control group, we included 25 healthy volunteers for the TPM measurements and 20 healthy volunteers for nT1 mapping with no known cardiovascular or other relevant systemic disease.

## Magnetic resonance imaging acquisition

**Fig. 1:**
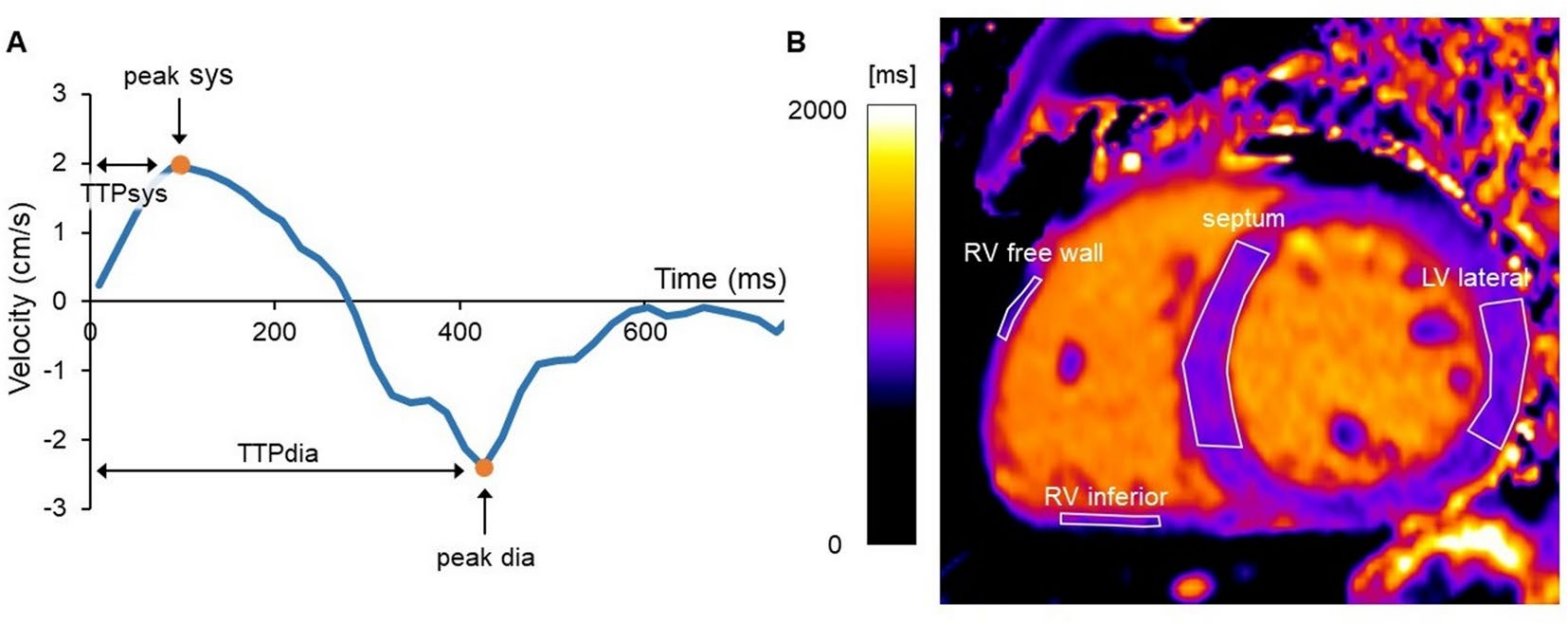
(**A**) Exemplary TPM velocity time course with labelling of the parameters peak systolic velocity (peak sys), peak diastolic velocity (peak dia), systolic time to peak (TTPsys), and diastolic time to peak (TTPdia). (**B**) Schematic of locations of native T1 mapping measurements on an exemplary image from a 29-year-old healthy volunteer.

Participants underwent a CMR examination using a 1.5 Tesla MRI-System (Avanto FIT, Siemens Healthineers, Erlangen, Germany). The MRI protocol comprised clinically indicated standard CMR sequences routinely performed, supplemented by additional study-specific sequences. In detail, the protocol consisted of the following sequences: (i) unenhanced ECG-synchronised Cine Magnetic Resonance Imaging (CINE)-steady-state free precession (SSFP) sequences in breath-hold technique in the 4-chamber,3-chamber-plane, and in-plane aligned to the LV and RV outflow tract (LVOT/RVOT); (ii) CINE-SSFP-sequences with continuous short-axis CINE-sequences from the atrioventricular ring to the apex and continuous CINE-sequences in the axial orientation covering the RV for the quantification of the LV and RV, respectively; (iii) unenhanced True Fast Imaging with Steady-State Precession (TRUFI) sequences in axial, coronal, and sagittal planes for anatomical visualisation of the thoracic organs; (iv) modified look-locker inversion recovery (MOLLI) sequences with a slice thickness of 8 mm for native and contrast-enhanced T1 mapping of the ventricles at 3 levels in the short-axis plane (apical, midventricular, basal); (v) LGE imaging in the short-axis plane, coronal plane, and 4-chamber view using inversion recovery gradient-echo sequences and image acquisition 10–15 min after the intravenous administration of a gadolinium-containing contrast agent (Prohance, Bracco Imaging Deutschland GmbH, Konstanz, Germany; dose adjusted according to patient weight); (vi) velocity-encoded phase-contrast imaging (TPM) for radial, long-axis, and circumferential systolic and diastolic peak velocities and time to peak (TTP) (Fig. 1A) at the ventricular base, midventricular, and apex-level^28^. Cine TPM images were reconstructed with a temporal resolution of 30–40 ms.

## Magnetic resonance data analysis

### Right and left ventricular volumes and ejection fraction

LV and RV volume and function were analysed by a radiologist using the medical platform syngo via (Siemens Healthineers GmbH, Erlangen, Germany). Volumetric assessment of the LV was performed semi-automatically using automatic segmentation of the endocardial borders in end-diastolic and end-systolic phases on short-axis images from apex to base, followed by manual correction. The same procedure was applied to the RV, using axial slices instead. The ventricular volumes and RV and LV EF were automatically calculated by the software. All measurements obtained via this method were internally validated by two radiologists certified in CMR.

EF in patients with rTOF was classified as normal, if the value lay above two standard deviations below the average EF of the healthy volunteers. Mean RV EF of the healthy volunteers was 60.5% with a standard deviation of 7.4%, and mean LV EF of the healthy volunteers was 63.7% with a standard deviation of 4.7%. Therefore, RV EF > 45.8% and LV EF > 54.4% was defined as normal.

## Tissue phase mapping

Analysis of TPM sequences was performed as reported previously by a custom-made software package implemented in MATLAB R2024a (Mathworks, Natick, MA, USA)^28^. In short, after semi-automated segmentation of RV and LV contours, the in-plane velocities of both ventricles were converted into velocities perpendicular and circumferential to the endocardium. Global and segmental radial (Vr), long-axis (Vz), and circumferential (Vφ) velocity time courses were used to derive systolic and diastolic peak velocities, as well as systolic and diastolic TTP (Fig. 1A). Division of the ventricles into segments was performed according to the 16-segment model of the American Heart Association^29^ and the 10-segment model described by Menza et al.^28^, for LV and RV, respectively, and values were averaged into base, middle, and apex for LV and RV for each patient.

### Native T1 mapping

Native T1 values were manually assessed in both the LV and RV using the radiology information and picture archiving and communication system DeepUnity (Dedalus Healthcare Group AG, Bonn, Germany). Regions of interest (ROIs) were placed manually on mid-ventricular short-axis T1 maps in the mid-myocardial layer in the interventricular septum, lateral LV wall, RVOT, anterior RV free wall and inferior RV wall (see Fig. 1B), maintaining predefined distances to both the endocardial and epicardial borders to minimize contamination by blood pool and epicardial fat. ROIs were slightly eroded to further reduce partial volume effects. Only motion-corrected MOLLI T1 maps were used, and all maps were visually inspected for fitting errors and artefacts; segments affected by insufficient image quality were excluded. ROI placement was reviewed by an experienced investigator to ensure consistency and robustness. Global nT1 values were assessed in the LV by segmentation of the entire myocardium at base, mid, and apical. Due to the thin wall of the RV, measurements were only performed when the wall thickness within the ROI exceeded 2.8 pixels (pixel size 1.41 mm).

## Late gadolinium enhancement

LGE analysis was conducted using syngo via (Siemens Healthineers GmbH, Erlangen, Germany). In the short-axis plane, coronal plane, and 4-chamber view, the presence and location of LGE were visually assessed by two radiologists in consensus. LGE was defined as hyperenhancement relative to the surrounding myocardium and categorized by location and pattern. Areas of artefact were carefully excluded from analysis.

### Statistical analysis

Statistical analyses were performed in OriginPro 2024b (OriginLab Corporation, Northampton, MA, USA). All values are presented as total numbers, medians, and interquartile ranges (Q3 – Q1, [IQR]), unless indicated otherwise. Due to non-normal distribution of several TPM, nT1, and volumetric parameters as assessed by Shapiro-Wilk tests, comparison between patients with rTOF and healthy volunteers was performed by Mann-Whitney U tests, and correlation analyses between TPM and nT1 values within the rTOF group by Spearman correlation. Dependency of the presence of patch material and the presence of LGE in the RVOT, and gender differences between the patient and control groups were calculated using Chi-squared test. *P*-values < 0.05 were considered as indicating statistical significance for all analyses.

## Results

### Characteristics of the study cohort

We examined 17 patients with rTOF (4 females) with a median age of 20 years (range 7–57 years) (Table 1). Upon clinical evaluation of the CMR, three of these patients (0 females, median age 32 years [range 32–44 years]) required operative pulmonary valve replacement (PVR) and then received a follow-up CMR 2–4 months after the operation. The remaining 14 patients did not require PVR (4 females, median age 18 years [range 7–57 years]). The control group for TPM and volumetric parameters comprised 25 healthy volunteers (12 females; median age 26 years [range 22–34 years]), and the control group for nT1 measurements 20 healthy volunteers (10 females; median age 34 years [range 29–44 years]). There was no difference in gender between the patient group and the control groups (rTOF vs. TPM control group: X^2^ = 2.18, *p* = 0.140; rTOF vs. nT1 control group: X^2^ = 3.66, *p* = 0.056). The control groups were slightly older than the rTOF group (rTOF vs. TPM control group: median 20 vs. 26 years, *U* = 106, *Z* = -2.736, *p* = 0.006; rTOF vs. nT1 control group: median 20 vs. 34 years, *U* = 54, *Z* = -3.534, *p* < 0.001).

**Table 1:** Demographic and clinical characteristics of the rTOF patient group.

Total patient group (*N*)	17
Age (median [range])	20 [7–57] years
Gender (N)	4 female / 13 male
Patients requiring PVR after CMR (N)	3
Number of past surgeries per patient (median [range])	1 [1–3]
Aortopulmonary shunt previous to operative repair	4
Type of operative repair (N): Transanular patch Valve-sparing repair Unknown	7 7 3
Other cardiovascular operations after repair (N): PVR PV reconstruction	3 1
Number of past catheter interventions per patient (median [range])	1 [0–4]
Type of past catheter interventions in the rTOF group (N): No past cath. Interventions RVOT or pulmonary artery intervention (BAP ± stent implantation) BVP of PV	11 5 1
History of atrial tachyarrhythmias (N)	2
History of ventricular tachyarrhythmias (N)	0

BAP, Balloon angioplasty; BVP, Balloon valvuloplasty; CMR, Cardiovascular magnetic resonance imaging; PV, Pulmonary valve; PVR, Pulmonary valve replacement; RVOT, Right ventricular outflow tract.

### Regional and global contractility (TPM and EF)

**Fig. 2:**
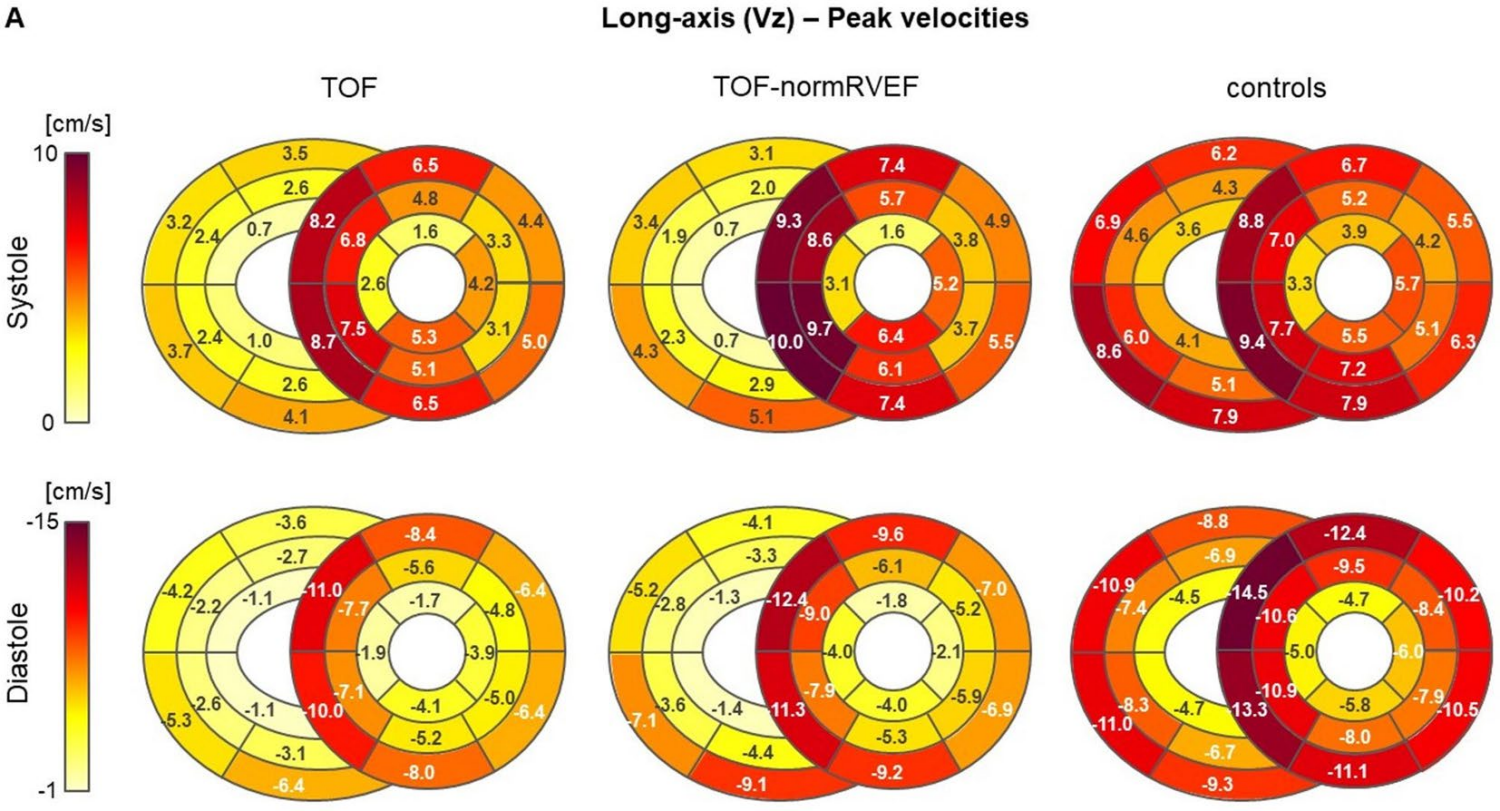

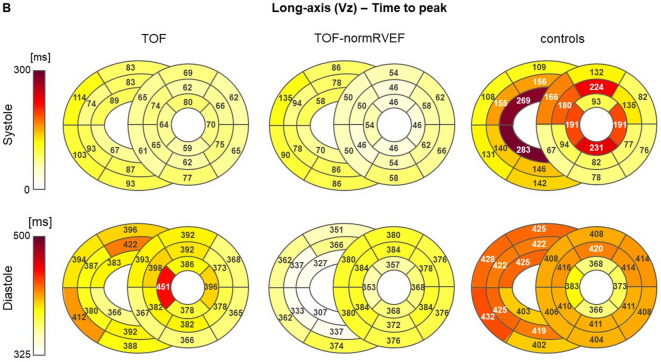
Segmental long-axis systolic and diastolic peak velocities (**A**) and time to peak (**B**) in patients with rTOF, patients with rTOF and normal right ventricular ejection fraction (‘TOF-normRVEF’), and in healthy volunteers (‘controls’).

For improved readability of this section, median values and IQR for each measurement and region including *U*- and *p*-values of the corresponding Mann-Whitney U test in patients with rTOF and healthy volunteers are reported in Table 2 and 3. Radial, long-axis, and circumferential systolic and diastolic peak velocities were smaller in all regions (base, middle, and apex) of both LV and RV in patients with rTOF compared to healthy volunteers, except for the radial peak systolic velocity at the RV apex and the circumferential peak diastolic velocity at the RV and LV apex (Table 2 and 3, Fig. 2). Long-axis and circumferential systolic TTP was shorter at base and middle of LV and RV in patients with rTOF versus controls (Table 2 and 3). Long-axis diastolic TTP was shorter, and circumferential diastolic TTP was longer in base and middle RV in patients with rTOF versus healthy controls (Table 2). There were no statistically significant differences in radial systolic and diastolic TTP in patients with rTOF and healthy volunteers in any region of the heart.

**Table 2:** Right ventricular tissue phase mapping parameters including peak systolic velocities, peak diastolic velocities, systolic time to peak, and diastolic time to peak for patients with rTOF (*N* = 17) and for healthy volunteers (‘controls’; *N* = 25).

		Radial (Vr)					Long axis (Vz)					Circumferential (Vφ)				
		rTOF		Controls		MW*	rTOF		Controls		MW*	rTOF		Controls		MW*
		med	IQR	med	IQR	*p*	med	IQR	med	IQR	*p*	med	IQR	med	IQR	*p*
Peak sys (cm/s)	base	2.6	0.8	4.1	0.7	** < 0.001**	3.4	1.4	7.1	1.7	** < 0.001**	-1.3	1.3	-3.5	1.2	** < 0.001**
	mid	2.6	0.8	3.8	0.8	** < 0.001**	2.3	1.2	4.2	2.0	** < 0.001**	-1.1	1.1	-3.0	1.2	** < 0.001**
	apex	2.5	0.8	2.9	1.1	0.056	0.9	0.9	3.0	1.5	** < 0.001**	-1.4	1.2	-3.0	1.5	** < 0.001**
Peak dia (cm/s)	base	-3.2	1.4	-5.2	0.9	** < 0.001**	-4.4	3.2	-9.5	2.7	** < 0.001**	0.9	1.6	3.5	1.5	** < 0.001**
	mid	-3.9	1.0	-4.9	0.8	**0.002**	-2.3	2.2	-6.9	1.8	** < 0.001**	1.2	1.5	2.4	1.3	** < 0.001**
	apex	-3.7	1.5	-4.4	1.1	**0.005**	-0.6	1.0	-4.0	0.9	** < 0.001**	2.0	1.3	2.1	1.2	0.343
TTP sys (ms)	base	151	80	138	25	0.493	80	60	160	107	**0.012**	30	10	330	300	** < 0.001**
	mid	171	70	160	43	0.231	70	41	160	128	**0.001**	30	0	53	21	** < 0.001**
	apex	111	90	138	64	0.167	70	60	309	256	** < 0.001**	70	50	53	21	0.672
TTP dia (ms)	base	394	90	385	57	0.583	374	80	415	43	**0.035**	404	257	138	21	** < 0.001**
	mid	394	80	415	64	0.678	384	73	428	21	**0.019**	342	153	160	256	**0.011**
	apex	394	54	437	43	0.066	394	243	437	43	0.278	342	111	309	277	0.399

*P-values given for Mann-Whitney U (MW) statistics comparing patients with rTOF with controls for each given parameter. Significant values are highlighted in bold script. *Med* median, *IQR* interquartile range, *peak dia* peak diastolic velocity, *peak sys* peak systolic velocity, *TTP dia* diastolic time to peak, *TTP sys* systolic time to peak.

**Table 3:** Left ventricular tissue phase mapping parameters including peak systolic velocities, peak diastolic velocities, systolic time to peak, and diastolic time to peak for patients with TOF (*N* = 17) and for healthy volunteers (‘controls’; *N* = 25).

		Radial (Vr)					Long axis (Vz)					Circumferential (Vφ)				
		TOF		Controls		MW*	TOF		*controls*		MW*	TOF		Controls		MW*
		med	IQR	med	IQR	*p*	med	IQR	med	IQR	*p*	med	IQR	med	IQR	*p*
Peak sys (cm/s)	*base*	2.2	0.7	2.8	0.6	** < 0.001**	5.3	3.0	7.0	1.5	**0.022**	-0.9	1.5	-3.0	1.2	** < 0.001**
	*mid*	2.4	0.6	2.8	0.3	**0.002**	4.6	1.5	5.6	1.1	**0.031**	-1.4	1.7	-3.9	1.3	**0.001**
	*apex*	1.9	0.6	2.5	0.4	**0.001**	3.0	2.3	4.0	2.1	0.095	-1.7	1.5	-3.4	0.9	** < 0.001**
Peak dia (cm/s)	*base*	-4.0	1.0	-4.7	1.0	**0.001**	-8.7	2.1	-11.1	2.6	** < 0.001**	0.8	0.8	2.6	1.0	** < 0.001**
	*mid*	-4.3	1.2	-5.2	0.7	**0.007**	-5.7	2.4	-8.8	2.9	** < 0.001**	1.2	1.4	2.1	0.8	**0.012**
	*apex*	-3.2	1.2	-5.0	0.8	** < 0.001**	-2.1	1.9	-4.6	2.0	** < 0.001**	2.1	1.2	2.3	0.7	0.698
TTP sys (ms)	*base*	101	50	117	43	0.062	60	50	53	21	0.193	30	30	53	23	**0.003**
	*mid*	101	90	117	21	0.298	50	40	72	21	**0.012**	30	20	53	0	**0.001**
	*apex*	111	40	96	21	0.274	50	40	53	21	**0.005**	50	40	53	0	0.297
TTP dia (ms)	*base*	384	80	394	43	0.511	394	84	406	43	0.341	424	123	160	170	** < 0.001**
	*mid*	404	80	415	43	0.278	404	80	415	43	0.398	384	157	309	298	0.462
	*apex*	414	90	428	30	0.778	394	90	394	43	0.799	358	87	351	64	0.799

*P-values given for Mann-Whitney U (MW) statistics comparing patients with rTOF with controls for each given parameter. Significant values are highlighted in bold script. *Med* median, *IQR* interquartile range, *peak dia* peak diastolic velocity, *peak sys* peak systolic velocity, *TTP dia* diastolic time to peak, *TTP sys* systolic time to peak.

Twelve of 17 patients with rTOF had reduced RV EF (i.e. < 45.8%, see Methods), and 5 of 17 patients with rTOF had a normal RV EF. Even in patients with normal RV EF, TPM showed reduced systolic and diastolic peak velocities and delayed TTP in the long-axis across all RV regions, as well as lower peak velocities and shorter systolic TTP in the circumferential direction (Table S1, Fig. 2).

Thirteen of 17 patients with rTOF had reduced LV EF (i.e. < 54.4%, see Methods), and the remaining 4 patients had a normal LV EF. In these patients with normal LV EF, LV TPM did not show any consistent differences compared to healthy controls in any region (Table S2).

Comparison of EF and volumetric parameters yielded significantly lower RV and LV EF in patients with rTOF compared to healthy volunteers (Table 4). RV end-diastolic and end-systolic volumes were significantly larger, and LV end-diastolic volume significantly smaller in patients with rTOF compared to healthy volunteers (Table 4).

**Table 4:** Volumetric data for the left and right ventricle of the patients with rTOF and the healthy volunteers (‘controls’).

Left ventricle							Right ventricle					
	rTOF		Control		Mann-W U*		rTOF		Control		Mann-W U*	
	med	IQR	med	IQR	U	*p*	med	IQR	med	IQR	U	*p*
EF (%)	51	5	63	5	9	< 0.001	39	13	62	9	11	< 0.001
EDV (ml/m^2^)	69	22	77	47	124	0.024	101	27	76	51	339	0.001
ESV (ml/m^2^)	33	16	27	16	287	0.058	64	30	31	24	402	< 0.001

*EDV* end-diastolic volume, *EF* ejection fraction, *ESV* end-systolic volume, *med* median, *IQR* interquartile range, *Mann-W U* Mann-Whitney U statistic. *Mann Whitney U statistic indicated for rTOF versus controls for each respective parameter.

There was no significant correlation between RV EF and LV EF in patients with rTOF (*r* = 0.463, *p* = 0.061) or in healthy volunteers (*r* = 0.001, *p* = 0.996). There was also no conclusive correlation between RV TPM parameters and RV EF or LV TPM parameters and LV EF in patients with rTOF or in healthy volunteers (Table S3).

There was no correlation between RV TPM parameters and pulmonary regurgitation fraction, however there was significant positive correlation between RV radial, circumferential, and diastolic time to peak and right ventricular end-diastolic volume (e.g. correlation of circumferential diastolic TTP at mid-level RV with RV end-diastolic volume: *r* = 0.719, *p* = 0.002).

### Myocardial fibrosis – native T1 mapping and late gadolinium enhancement

**Fig. 3:**
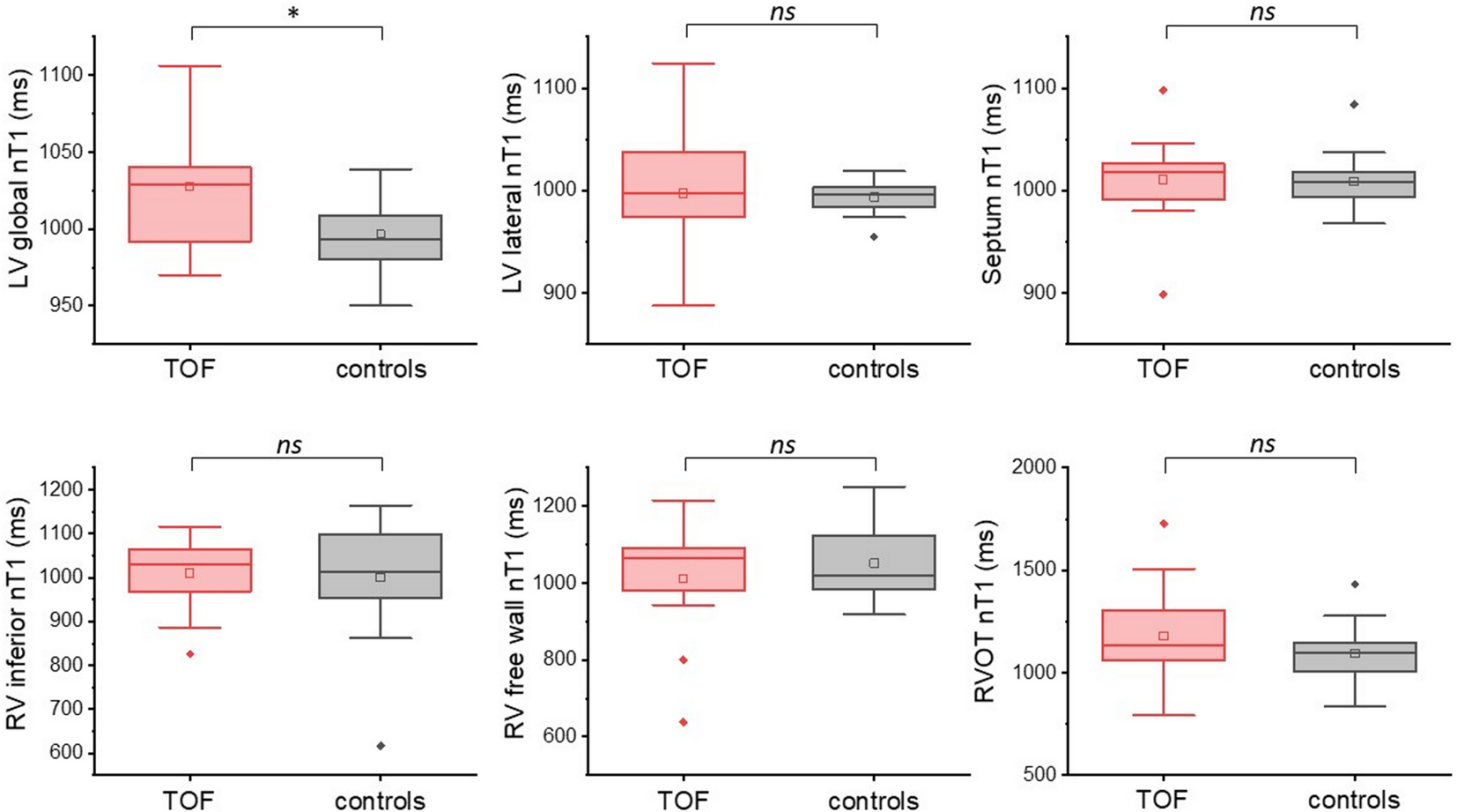
Native T1 times in different left and right ventricular regions for patients with rTOF and healthy volunteers (‘controls’). *LV* left ventricle, *nT1* native T1 time, *RV* right ventricle, *RVOT* right ventricular outflow tract, *TOF* tetralogy of Fallot.

RV wall thickness was sufficient for nT1 measurement in (i) the RVOT in 18 controls and 17 patients, (ii) the RV free wall in 16 controls and 12 patients, and (iii) inferior RV in 19 controls and 15 patients. Native T1 values were significantly higher in patients with rTOF than in controls in global LV (median [IQR] 1029 [48] ms vs. 993 [28] ms, *U* = 225, *Z* = 2.48, *p* = 0.013), but not in any other LV or RV regions (Fig. 3, Table S4).

In 16 of the 17 patients intravenous contrast agent was administered. LGE data was obtained in the LV of 13 patients and in the RV of 12 patients with rTOF; in two patients no LGE sequences were acquired, while motion artefacts obscured analysis in the LV of two and in the RV of three patients. Eight patients (8/12) demonstrated LGE in the RVOT. Only one patient with rTOF demonstrated additional LGE in other RV regions and in the LV. Neither LGE nor nT1 in the RVOT were associated with transanular patch implantation (LGE X^2^ = 3.12, *p* = 0.538; nT1: median [IQR] 1135 [416] vs. 1146 [658], *U* = 20, *Z* = -0.511, *p* = 0.609). Patients with the presence of LGE in the RVOT demonstrated higher nT1 in the RVOT than patients without LGE in the RVOT (median [IQR] 1078 [305] vs. 791 [244.5] ms, *U* = 2, *Z* = 2.293, *p* = 0.022).

### Correlation of segmental contractility (TPM) and fibrosis (nT1)

**Fig. 4:**
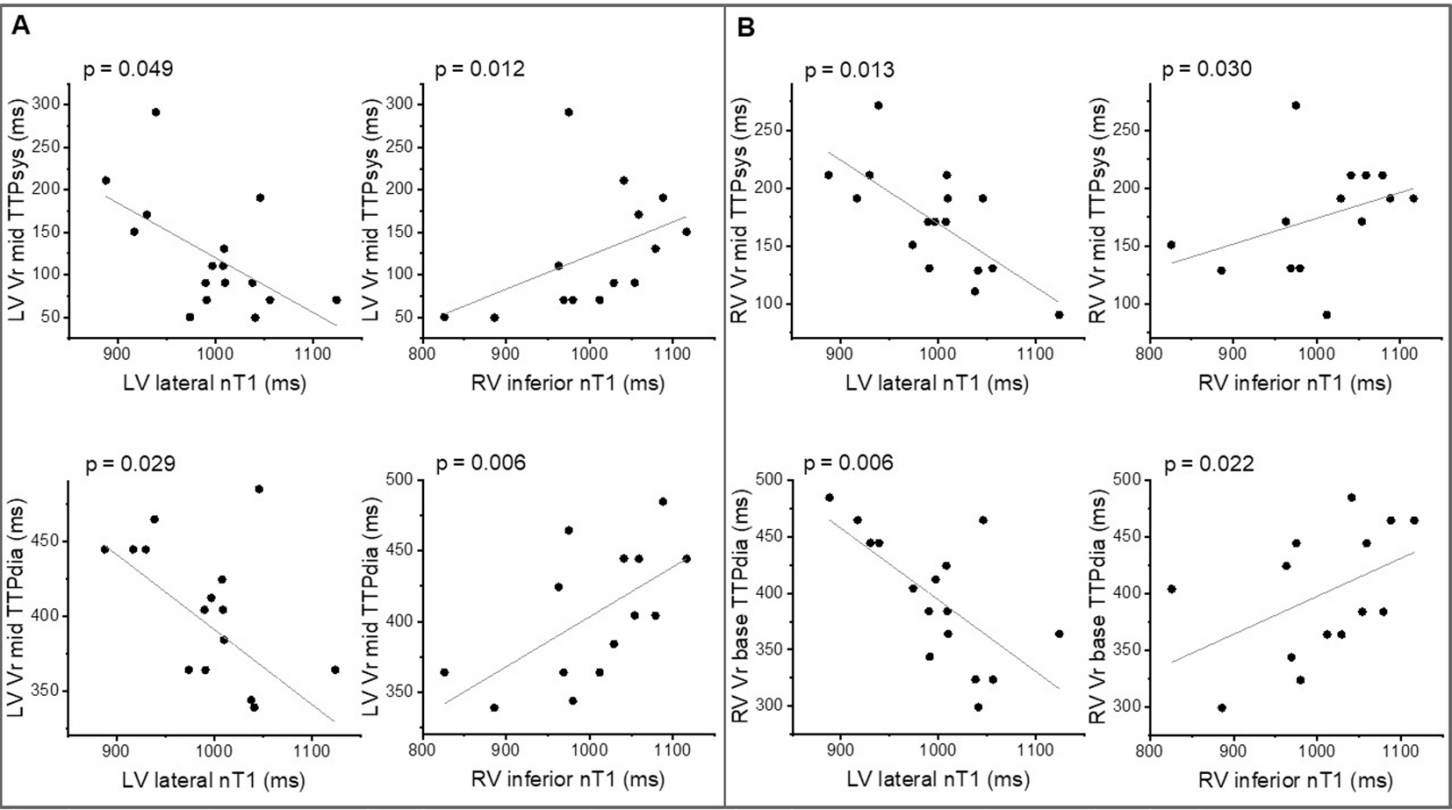
Examples of significant correlations between native T1 times and radial time to peak of the left ventricle (A) and the right ventricle (B) in the rTOF patient group. *LV* left ventricle, *nT1* native T1 time, *RV* right ventricle, *TTPdia* diastolic time to peak, *TTPsys* systolic time to peak, *Vr* radial.

Spearman correlation revealed a significant association of increased nT1 in LV lateral with especially shorter radial, long-axis and circumferential diastolic TTP in LV and RV regions (Table 5; Fig. 4). Increased nT1 in RV inferior was significantly positively correlated with longer systolic and diastolic LV and RV TTP (Table 5). No other nT1 regions were consistently correlated with segmental contractility parameters measured by TPM (Table S5a and S5b).

**Table 5:** Spearman correlations of systolic and diastolic time to peak with native T1 times in LV lateral and RV inferior in the rTOF patient group.

	Native T1 mapping												
					LV lateral		RV inferior			LV lateral		RV inferior	
					*r*	*p*	*r*	*p*		*r*	*p*	*r*	*p*
Tissue phase mapping	*radial (Vr)*	TTP sys (ms)	base	Left ventricle	-0.239	0.373	**0.733**	**0.003**	Right ventricle	-0.491	0.053	0.358	0.209
			mid		**-0.499**	**0.049**	**0.648**	**0.012**		**-0.604**	**0.013**	**0.580**	**0.030**
			apex		-0.396	0.129	**0.687**	**0.007**		-0.431	0.096	0.391	0.167
		TTP dia (ms)	base		**-0.526**	**0.036**	**0.662**	**0.010**		**-0.657**	**0.006**	**0.605**	**0.022**
			mid		**-0.545**	**0.029**	**0.695**	**0.006**		**-0.601**	**0.014**	**0.750**	**0.002**
			apex		**-0.534**	**0.033**	**0.640**	**0.014**		-0.449	0.081	**0.742**	**0.002**
	*long axis (Vz)*	TTP sys (ms)	base		-0.409	0.116	0.528	0.052		-0.442	0.087	**0.635**	**0.015**
			mid		-0.486	0.056	**0.719**	**0.004**		-0.436	0.091	**0.625**	**0.017**
			apex		-0.408	0.117	0.441	0.114		-0.147	0.587	**0.550**	**0.042**
		TTP dia (ms)	base		**-0.524**	**0.037**	**0.650**	**0.012**		-0.397	0.127	**0.760**	**0.002**
			mid		-0.448	0.082	**0.608**	**0.021**		**-0.520**	**0.039**	**0.592**	**0.026**
			apex		**-0.534**	**0.033**	0.263	0.363		-0.432	0.095	0.452	0.105
	*circumfer. (Vφ)*	TTP sys (ms)	base		-0.134	0.620	0.496	0.072		-0.021	0.937	0.342	0.231
			mid		-0.138	0.610	**0.570**	**0.033**		-0.033	0.904	0.347	0.224
			apex		-0.221	0.411	**0.691**	**0.006**		-0.150	0.578	0.306	0.287
		TTP dia (ms)	base		-0.236	0.379	0.209	0.473		-0.259	0.332	0.260	0.370
			mid		-0.328	0.215	0.490	0.076		**-0.523**	**0.038**	0.493	0.073
			apex		**-0.542**	**0.030**	0.435	0.120		**-0.642**	**0.007**	0.413	0.142

*LV* left ventricle, *peak sys* peak systolic velocity, *peak dia* peak diastolic velocity, *RV* right ventricle, *T1M* T1 mapping, *TPM* tissue phase mapping, *TTP dia* diastolic time to peak, *TTP sys* systolic time to peak. Significant correlations are indicated in bold script.

### Patients with pulmonary valve replacement

Three patients required PVR after CMR. Those patients were significantly older (34 [13] vs. 18 [5] years, *U* = 39, *p* = 0.027), had significantly larger RV size (RVEDV: 174 [19] vs. 96.5 [18] ml/m², *U* = 42, *p* = 0.010; RVESV: 107 [4] vs. 60 [30] ml/m², *U* = 42, *p* = 0.010), and a significantly higher pulmonary regurgitant fraction (52 [1] vs. 29 [19.5] %, *U* = 41.5, *p* = 0.012) in their pre-operative CMR compared to those patients not requiring PVR. nT1 and TPM parameters did not differ between patients with and without PVR.

RV end-diastolic volume was not significantly correlated with nT1 values in the entire rTOF group, but demonstrated significant positive association with systolic circumferential LV peak velocities especially at the base (*r* = 0.812, *p* = 0.000), but also at mid-ventricular and apical level (*r* = 0.762, *p* = 0.001 and *r* = 0.579, *p* = 0.019, respectively).

### Pre- versus post-operative CMR

For comparison of pre- versus post-operative CMR results in patients with PVR, see Supplementary Materials.

## Discussion

In our prospective study on 17 patients with rTOF, we examined potential links between diffuse, non-scar related fibrosis and regional mechanical (i.e. contractile) function using CMR. Main findings of our study were.

1. Pathological systolic and diastolic function as identified by segmental biventricular TPM,
2. Altered contractile RV function even in patients with normal RV EF,
3. Diffuse myocardial fibrosis, but no LGE, especially in the LV, and.
4. Association of regional contractility with diffuse fibrosis in both ventricles in patients with rTOF.

Our biventricular TPM sequences described above and in Menza et al.^28^ revealed a reduction in regional radial, long-axis and circumferential systolic and diastolic peak velocities, shortened radial and altered TTP in both ventricles of patients with rTOF. With all three levels (base, mid and apex) of the ventricles affected, this indicates diffuse contractile impairment of the myocardium, consistent with the decreased biventricular EF in the rTOF group and with previous strain-based investigations^21,22,30^. However, in our study, even those patients with normal RV EF demonstrated impaired regional velocities in the RV, whereas patients with normal LV EF did not demonstrate impaired regional contractility in the LV. This finding may indicate a higher sensitivity of TPM in patients with TOF for the diagnosis of impaired mechanical RV function and may identify RV dysfunction earlier than conventional functional analyses based on EF or strain^31,32^. In addition, both increased RV end-diastolic volume and increased nT1 in the inferior RV were associated with longer diastolic time to peak, indicating that increased RV remodelling also leads to decreased diastolic function.

As expected, LGE was frequently present in the RVOT of patients with rTOF (in 67%), presumably as a result of previous operative interventions, but without relation to potential patch material in the RVOT. In line with this, patients exhibiting LGE in the RVOT also demonstrated abnormal nT1 values, suggestive of underlying fibrosis. In the entire patient group, diffuse fibrosis (i.e. nT1) was only statistically significantly increased in global LV, but not in segmental analysis when compared to the control group (Fig. 3, Tab. S4). The latter may have been due to power issues and warrants further investigation in larger cohorts. Increased global LV nT1 may explain the impaired LV function demonstrated in our patient group, as increased fibrosis leads to stiffer myocardium with altered mechanical properties^33^. The patients with increased global LV nT1 showed no LGE in this area, indicating successful detection of diffuse fibrotic myocardial changes without the necessity of administration of intravenous contrast agent, consistent with previous reports^34^. Finally, this points towards LV involvement in what was for a long time considered to be an ‘RV disease’ and highlights the necessity for further in-depth LV evaluation.

Unexpectedly, we found differential mechanical remodelling in response to diffuse fibrosis in RV and LV, with significant associations of shorter biventricular TTP with increased nT1 in the lateral LV, and of longer biventricular TTP with increased nT1 in the inferior RV. On one hand, increased fibrosis may result in less displacement overall of the myocardium, therefore leading to shorter TTP, on the other, increased fibrosis may slow myocardial motion, leading to longer TTP during contraction and relaxation if displacement is achieved despite fibrosis. Our results may therefore reflect the interplay between different factors affecting mechanical myocardial properties, such as wall thickness, cardiomyocyte orientation and (an)isotropy, as well as fibrosis organisation (e.g. more focal/patchy/perivascular fibrosis vs. diffuse/interstitial fibrosis), all of which may be significantly different in the RV and LV^35–37^. In addition, interventricular dependence on the basis of enlarged RV with abnormal contractile patterns may play a role, as larger RV size of those patients requiring PVR was also associated with altered circumferential peak velocities of the LV. Alternatively, these results may also reflect an inherent reaction of each ventricle to adapt to increased fibrosis. This remains unclear and requires further investigation, both regarding organ-scale MRI-based evaluation and, ideally, also tissue-based comparison of LV and RV remodelling of the myocardium itself.

### Limitations

The small sample size in our study warrants cautious interpretation of our results and requires larger cohort investigations to enable extrapolation to different TOF subpopulations. When investigating patients with congenital heart disease, it must be kept in mind that these patient groups, despite a common diagnosis of a specific disease, are often highly heterogeneous in their anatomy and haemodynamics. This considerably complicates the interpretation of causality in myocardial pathology.

The healthy volunteers for the nT1 and the TPM measurements were slightly but significantly older than the rTOF patients. However, when considering the absolute values, this is presumably not biologically relevant, as previous studies have not demonstrated significant changes in nT1 or TPM parameters over 10–20 years^38,39^, and, in our study, neither patients nor healthy volunteers demonstrated conclusive correlations between age and TPM parameters (Tab. S6). In addition, it was not possible to correlate nT1 and TPM values in healthy subjects in this study. In light of lacking other studies investigating this, evaluation of links between nT1 and TPM in subjects without cardiac disease is urgently required.

Regarding limitations of our MRI protocol, despite application of customised TPM sequences for measurements of myocardial contractility, these have been applied widely and shown robust results^28,40,41^. The temporal resolution of the tissue phase mapping sequence (30–40 ms) may lead to some temporal smoothing and potential underestimation of sharp peak velocities, particularly during early diastole. However, this resolution is in line with prior TPM studies and is sufficient for robust group-level comparisons and assessment of global and regional myocardial motion^42^. Finally, extracellular volume was not calculated from T1 measurements, as serum haematocrit values within 24 h of imaging were not available for our cohort (as would be required for extracellular volume calculation). Given the small sample size and the considerable variability associated with calculating synthetic extracellular volume^43^, we chose not to use synthetic haematocrit in this study.

## Conclusion

Patients with rTOF demonstrate impaired myocardial function both in the systolic and the diastolic phase of the contractile cycle. TPM may be a valuable tool to help early detection especially of RV dysfunction in patients with rTOF, before EF is affected. In addition, diffuse fibrosis seems to have a different effect on RV and LV contraction which highlights the need to explore ventricle-specific myocardial mechanisms and the role of ventricular interdependence.

## Supplementary Information

Below is the link to the electronic supplementary material.

Supplementary Material 1

## Data Availability

The datasets underlying this study can be provided in anonymised form by the authors upon reasonable request.
