# Supplementary material for: Detecting early structural and functional myocardial alterations in patients with repaired tetralogy of fallot: a prospective MRI study
Source: Sci Rep. 2026 May 6;16:18521. doi: 10.1038/s41598-026-50982-5 (PMC13269691; doi:10.1038/s41598-026-50982-5)
Supplement: Supplementary file 1 — Supplementary Material 1 [file 41598_2026_50982_MOESM1_ESM.docx]

# Supplementary Material

to the manuscript entitled “Detecting Early Structural and Functional Myocardial Alterations in Patients with Repaired Tetralogy of Fallot: A Prospective MRI Study” by H.E. Kappler et al.

## Supplementary Results

Pre- versus post-operative MRI

Due to the small number of patients requiring PVR as result of the first CMR (N = 3, one patient no pre-operative TPM sequences), analysis of these data remains descriptive only. The three patients received their follow-up CMR 2.5, 3.5, and 4.5 months after PVR, respectively. Post-operatively all patients demonstrated smaller normalised RVEDV, RVESV, and RVSV, as well as reduced pulmonary regurgitant fraction (Fig. S1). There was no difference in global RV or LV function, as expressed by EF. TPM revealed smaller peak systolic velocities in long-axis of the LV at mid and apex, as well as in the RV (radial at the base, circumferential at base and apex), and smaller peak diastolic velocities in the RV (radial base, long-axis base and mid). However, these results do not indicate a systematic effect of PVR on segmental systolic or diastolic contractility in either ventricle. Finally, nT1 values in the septum were lower in the post-operative follow-up compared to pre-operatively (Fig. S1). While our patient numbers are too small to allow any conclusions transferable to a larger population, they do not indicate any striking improvement of RV function in our patients on short-term follow-up, while myocardial fibrotic remodelling may be reversible, in parts.


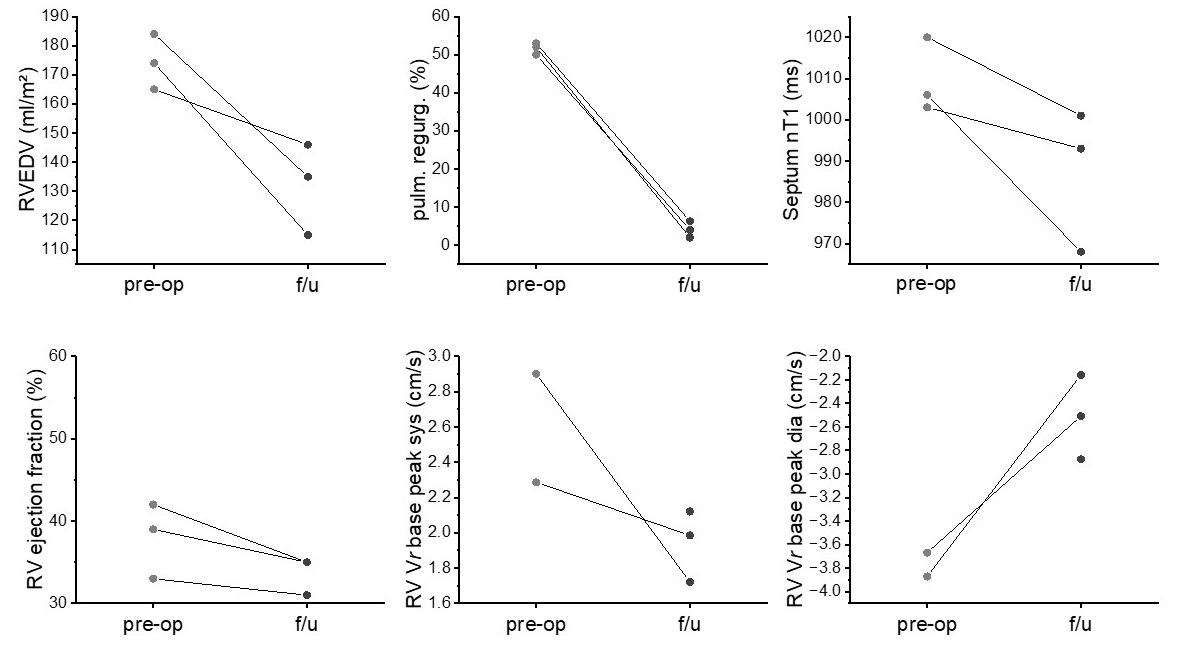


**Fig. S1:** Paired data on right ventricular size, function, and fibrosis in the three patients with rTOF from CMR before and several months after operative pulmonary valve replacement. *RV* right ventricle, *RVEDV* right ventricular end-diastolic volume, *pulm. regurg.* pulmonary regurgitant fraction, *nT1* native T1 time, *Vr* radial, *sys* systolic, *dia* diastolic, *pre-op* pre-operatively, *f/u* upon follow-up

## Supplementary Tables

**Tab. S1:** Right ventricular tissue phase mapping including parameters peak systolic velocities, peak diastolic velocities, systolic time to peak, and diastolic time to peak in patients with **rTOF and normal right ventricular ejection fraction** (TOF-normRVEF, N = 5).

|  | | **Radial (Vr)** | | | **Long axis (Vz)** | | | **Circumferential (Vφ)** | | |
| --- | --- | --- | --- | --- | --- | --- | --- | --- | --- | --- |
|  |  | *med* | *IQR* | *p** | *med* | *IQR* | *p** | *med* | *IQR* | *p** |
| **Peak sys** | *base* | 3.1 | 1.2 | **0.019** | 3.9 | 0.5 | **0.003** | -1.6 | 1.7 | **0.001** |
| *(cm/s)* | *mid* | 2.8 | 1.4 | 0.085 | 2.3 | 0.6 | **0.003** | -1.6 | 0.4 | **0.002** |
|  | *apex* | 2.7 | 1.3 | 0.867 | 0.4 | 0.6 | **0.002** | -1.6 | 0.1 | **0.012** |
| **Peak dia** | *base* | -4.6 | 1.1 | 0.133 | -5.5 | 3.0 | **0.026** | 1.6 | 0.8 | **0.002** |
| *(cm/s)* | *mid* | -4.7 | 0.7 | 1.000 | -3.3 | 1.2 | **0.001** | 1.3 | 0.6 | **0.023** |
|  | *apex* | -4.1 | 0.7 | 0.075 | -1.4 | 0.7 | **0.001** | 2.5 | 0.6 | 0.404 |
| **TTP sys** | *base* | 111 | 80 | 0.693 | 70 | 20 | **0.044** | 30 | 0 | **0.002** |
| *(ms)* | *mid* | 191 | 80 | 0.397 | 70 | 20 | **0.010** | 30 | 0 | **0.006** |
|  | *apex* | 90 | 80 | 0.130 | 70 | 40 | **0.009** | 70 | 0 | 0.378 |
| **TTP dia** | *base* | 364 | 20 | 0.538 | 364 | 20 | **0.032** | 384 | 121 | **0.010** |
| *(ms)* | *mid* | 384 | 20 | 0.615 | 364 | 20 | **0.004** | 304 | 93 | 0.237 |
|  | *apex* | 384 | 20 | 0.073 | 271 | 133 | **0.040** | 324 | 80 | 0.911 |

*P-values given for Mann-Whitney U statistic comparing TOF-normRVEF with healthy volunteers for each given parameter; significant differences are highlighted in bold script. *Med* median, *IQR* interquartile range, *peak dia* peak diastolic velocity, *peak sys* peak systolic velocity, *TPM* tissue phase mapping, *TTP sys* systolic time to peak, *TTP dia* diastolic time to peak.

**Tab. S2:** Left ventricular tissue phase mapping parameters including peak systolic velocities, peak diastolic velocities, systolic time to peak, and diastolic time to peak in patients with **rTOF and normal left ventricular ejection fraction** (TOF-normLVEF, N = 4).

|  | | **Radial (Vr)** | | | **Long axis (Vz)** | | | **Circumferential (Vφ)** | | |
| --- | --- | --- | --- | --- | --- | --- | --- | --- | --- | --- |
|  |  | *med* | *IQR* | *p** | *med* | *IQR* | *p** | *med* | *IQR* | *p* |
| **Peak sys** | *base* | 2.1 | 1.3 | **0.034** | 8.8 | 5.0 | 0.552 | -2.6 | 4.0 | 0.603 |
| *(cm/s)* | *mid* | 2.9 | 1.1 | 0.882 | 7.7 | 4.0 | 0.504 | -3.1 | 4.0 | 0.656 |
|  | *apex* | 2.6 | 1.1 | 0.882 | 4.5 | 1.3 | 0.766 | -2.5 | 2.3 | 0.158 |
| **Peak dia** | *base* | -4.7 | 2.5 | 0.458 | -9.0 | 4.9 | 0.334 | 0.9 | 1.7 | **0.031** |
| *(cm/s)* | *mid* | -4.0 | 1.9 | 0.265 | -6.5 | 4.3 | 0.102 | 1.5 | 2.9 | 0.458 |
|  | *apex* | -4.3 | 1.5 | 0.063 | -2.7 | 2.2 | **0.031** | 3.5 | 2.4 | 0.181 |
| **TTP sys** | *base* | 90 | 61 | 0.067 | 50 | 41 | 0.055 | 30 | 41 | 0.129 |
| *(ms)* | *mid* | 90 | 121 | 0.441 | 50 | 41 | **0.048** | 30 | 41 | 0.244 |
|  | *apex* | 90 | 41 | 0.109 | 50 | 41 | 0.099 | 49 | 40 | 0.238 |
| **TTP dia** | *base* | 364 | 125 | 0.409 | 364 | 125 | 0.295 | 444 | 145 | 0.068 |
| *(ms)* | *mid* | 384 | 105 | 0.369 | 364 | 85 | 0.133 | 304 | 125 | 0.823 |
|  | *apex* | 384 | 106 | 0.368 | 344 | 105 | 0.410 | 344 | 105 | 0.654 |

*P-values given for Mann-Whitney U statistic comparing TOF-normLVEF with healthy volunteers for each given parameter; significant differences are highlighted in bold script. *Med* median, *IQR* interquartile range, *peak dia* peak diastolic velocity, *peak sys* peak systolic velocity, *TPM* tissue phase mapping, *TTP sys* systolic time to peak, *TTP dia* diastolic time to peak.

**Tab. S3:** Spearman correlation analysis between ejection fraction and tissue phase mapping parameters of right and left ventricle in patients with rTOF and in healthy volunteers (‘controls’).

|  |  |  |  | **Ejection fraction** | | | | | | | | | |
| --- | --- | --- | --- | --- | --- | --- | --- | --- | --- | --- | --- | --- | --- |
|  |  |  |  |  | *Right ventricle* | | | |  | *Left ventricle* | | | |
|  |  |  |  |  | *TOF* | | *controls* | |  | *TOF* | | *controls* | |
|  |  |  |  |  | *r* | *p* | *r* | *p* |  | *r* | *p* | *r* | *p* |
| **Tissue phase mapping** | *Radial (Vr)* | *peak sys* | *base* | *Right ventricle* | -0.330 | 0.212 | 0.073 | 0.729 | *Left ventricle* | -0.221 | 0.410 | -0.324 | 0.114 |
|  |  |  | *mid* |  | 0.221 | 0.411 | 0.037 | 0.861 |  | 0.401 | 0.124 | -0.287 | 0.164 |
|  |  |  | *apex* |  | -0.038 | 0.889 | 0.257 | 0.214 |  | -0.168 | 0.533 | -0.385 | 0.057 |
|  |  | *peak dia* | *base* |  | -0.436 | 0.091 | -0.276 | 0.182 |  | -0.312 | 0.240 | 0.346 | 0.091 |
|  |  |  | *mid* |  | -0.130 | 0.632 | -0.071 | 0.736 |  | -0.193 | 0.474 | -0.021 | 0.921 |
|  |  |  | *apex* |  | -0.176 | 0.515 | -0.177 | 0.397 |  | -0.173 | 0.523 | -0.038 | 0.856 |
|  |  | *TTP sys* | *base* |  | 0.407 | 0.118 | -0.013 | 0.951 |  | -0.161 | 0.552 | -0.006 | 0.978 |
|  |  |  | *mid* |  | -0.323 | 0.222 | -0.032 | 0.879 |  | -0.376 | 0.151 | -0.124 | 0.554 |
|  |  |  | *apex* |  | -0.480 | 0.060 | -0.237 | 0.253 |  | -0.162 | 0.549 | -0.319 | 0.121 |
|  |  | *TTP dia* | *base* |  | -0.520 | **0.039** | -0.180 | 0.390 |  | -0.116 | 0.668 | 0.330 | 0.107 |
|  |  |  | *mid* |  | -0.242 | 0.367 | -0.037 | 0.861 |  | -0.120 | 0.657 | 0.012 | 0.956 |
|  |  |  | *apex* |  | 0.451 | 0.080 | -0.162 | 0.440 |  | 0.370 | 0.158 | 0.165 | 0.431 |
|  | *Long-axis (Vz)* | *peak sys* | *base* |  | -0.138 | 0.612 | 0.317 | 0.123 |  | -0.123 | 0.649 | -0.159 | 0.447 |
|  |  |  | *mid* |  | -0.221 | 0.411 | 0.116 | 0.582 |  | 0.355 | 0.177 | -0.130 | 0.535 |
|  |  |  | *apex* |  | -0.271 | 0.310 | 0.260 | 0.210 |  | -0.289 | 0.277 | -0.375 | 0.064 |
|  |  | *peak dia* | *base* |  | -0.185 | 0.492 | -0.280 | 0.176 |  | 0.103 | 0.704 | 0.200 | 0.339 |
|  |  |  | *mid* |  | -0.083 | 0.761 | -0.038 | 0.858 |  | -0.141 | 0.601 | -0.147 | 0.484 |
|  |  |  | *apex* |  | -0.373 | 0.155 | -0.283 | 0.181 |  | -0.076 | 0.779 | 0.008 | 0.971 |
|  |  | *TTP sys* | *base* |  | 0.354 | 0.179 | -0.018 | 0.932 |  | 0.438 | 0.090 | -0.217 | 0.298 |
|  |  |  | *mid* |  | -0.247 | 0.357 | 0.177 | 0.397 |  | -0.086 | 0.753 | 0.015 | 0.942 |
|  |  |  | *apex* |  | -0.340 | 0.197 | 0.002 | 0.993 |  | -0.024 | 0.931 | 0.220 | 0.291 |
|  |  | *TTP dia* | *base* |  | -0.161 | 0.552 | -0.247 | 0.233 |  | 0.168 | 0.534 | 0.199 | 0.341 |
|  |  |  | *mid* |  | -0.336 | 0.204 | -0.045 | 0.830 |  | -0.276 | 0.300 | 0.081 | 0.699 |
|  |  |  | *apex* |  | -0.413 | 0.112 | 0.195 | 0.351 |  | 0.522 | **0.038** | 0.044 | 0.836 |
|  | *Circumferential (Vφ)* | *peak sys* | *base* |  | -0.201 | 0.454 | 0.034 | 0.872 |  | -0.011 | 0.967 | -0.272 | 0.189 |
|  |  |  | *mid* |  | -0.466 | 0.069 | -0.137 | 0.513 |  | -0.535 | **0.033** | 0.234 | 0.260 |
|  |  |  | *apex* |  | 0.075 | 0.784 | -0.182 | 0.385 |  | 0.157 | 0.561 | 0.176 | 0.400 |
|  |  | *peak dia* | *base* |  | 0.075 | 0.782 | 0.132 | 0.530 |  | 0.003 | 0.991 | -0.268 | 0.194 |
|  |  |  | *mid* |  | 0.576 | **0.019** | 0.014 | 0.947 |  | 0.592 | **0.016** | -0.319 | 0.121 |
|  |  |  | *apex* |  | -0.381 | 0.145 | -0.201 | 0.336 |  | -0.153 | 0.572 | -0.021 | 0.920 |
|  |  | *TTP sys* | *base* |  | -0.274 | 0.304 | -0.203 | 0.330 |  | -0.212 | 0.430 | 0.063 | 0.766 |
|  |  |  | *mid* |  | 0.024 | 0.929 | -0.560 | **0.004** |  | -0.067 | 0.806 | 0.037 | 0.862 |
|  |  |  | *apex* |  | 0.234 | 0.382 | -0.314 | 0.126 |  | 0.304 | 0.253 | 0.168 | 0.421 |
|  |  | *TTP dia* | *base* |  | 0.218 | 0.417 | -0.478 | **0.016** |  | 0.296 | 0.265 | -0.005 | 0.983 |
|  |  |  | *mid* |  | -0.252 | 0.347 | -0.367 | 0.071 |  | -0.123 | 0.651 | -0.135 | 0.519 |
|  |  |  | *apex* |  | -0.386 | 0.140 | 0.023 | 0.912 |  | -0.329 | 0.214 | 0.397 | 0.050 |

*Peak dia* peak diastolic velocity, *peak sys* peak systolic velocity, *TTP dia* diastolic time to peak, *TTP sys* systolic time to peak. Statistically significant values are indicated in bold script.

**Tab. S4:** Native T1 values in different left and right ventricular regions in patients with rTOF and in healthy volunteers (‘controls’), as well as comparison between the groups with Mann Whitney U statistics.

|  | **TOF** | | | **control** | | | **Mann Whitney U** | | |
| --- | --- | --- | --- | --- | --- | --- | --- | --- | --- |
|  | *native T1 (ms)* | | | *native T1 (ms)* | | |  |  |  |
|  | *med* | *IQR* | *range (min-max)* | *med* | *IQR* | *range (min-max)* | *U* | *Z* | *p* |
| **LV global** | 1029 | 48 | 970−1106 | 993 | 28 | 950−1039 | 225 | 2.48 | **0.013** |
| **Septum** | 1018 | 35 | 899−1098 | 1008 | 25 | 968−1084 | 190.5 | 0.61 | 0.542 |
| **LV lateral** | 997 | 64 | 888−1956 | 997 | 19 | 955−1019 | 187.5 | 0.52 | 0.604 |
| **RVOT** | 1135 | 246 | 791−1728 | 1097 | 136 | 836−1431 | 186 | 1.07 | 0.283 |
| **RV free wall** | 1066 | 112 | 638−1215 | 1020 | 140 | 919−1250 | 97 | -0.29 | 0.776 |
| **RV inferior** | 1029 | 95 | 826−1116 | 1014 | 144 | 617−1164 | 139.5 | -0.09 | 0.931 |

*IQR* interquartile range, *LV* left ventricle, *RV* right ventricle, *RVOT* right ventricular outflow tract.

**Tab. S5a:** Spearman correlations between radial, long-axis, and circumferential tissue phase mapping and native T1 mapping in the right ventricle.

|  |  |  |  | **Native T1 mapping** | | | | | | | | | | | |
| --- | --- | --- | --- | --- | --- | --- | --- | --- | --- | --- | --- | --- | --- | --- | --- |
|  |  |  |  | *LV global* | | *Septum* | | *LV lateral* | | *RVOT* | | *RV free wall* | | *RV inferior* | |
|  |  |  |  | *r* | *p* | *r* | *p* | *r* | *p* | *r* | *p* | *r* | *p* | *r* | *p* |
| **Tissue phase mapping - right ventricle** | *Radial (Vr)* | *peak sys* | *base* | -0.226 | 0.436 | -0.182 | 0.499 | 0.371 | 0.158 | 0.232 | 0.387 | 0.137 | 0.655 | -0.288 | 0.318 |
|  |  |  | *mid* | -0.319 | 0.267 | -0.346 | 0.189 | 0.094 | 0.729 | 0.203 | 0.451 | -0.170 | 0.578 | -0.033 | 0.911 |
|  |  |  | *apex* | -0.231 | 0.427 | -0.172 | 0.524 | 0.303 | 0.254 | -0.029 | 0.914 | -0.115 | 0.707 | 0.121 | 0.681 |
|  |  | *peak dia* | *base* | 0.007 | 0.982 | -0.078 | 0.774 | -0.300 | 0.259 | -0.121 | 0.656 | -0.231 | 0.448 | -0.174 | 0.553 |
|  |  |  | *mid* | 0.200 | 0.493 | 0.094 | 0.729 | 0.118 | 0.664 | -0.074 | 0.787 | -0.269 | 0.374 | 0.077 | 0.794 |
|  |  |  | *apex* | 0.160 | 0.584 | 0.188 | 0.485 | -0.156 | 0.564 | -0.212 | 0.431 | -0.055 | 0.859 | -0.393 | 0.164 |
|  |  | *TTP sys* | *base* | 0.185 | 0.527 | 0.067 | 0.806 | -0.491 | 0.053 | -0.129 | 0.633 | -0.011 | 0.971 | 0.358 | 0.209 |
|  |  |  | *mid* | 0.111 | 0.706 | -0.024 | 0.930 | **-0.604** | **0.013** | -0.092 | 0.735 | 0.083 | 0.787 | **0.580** | **0.030** |
|  |  |  | *apex* | 0.187 | 0.521 | -0.386 | 0.139 | -0.431 | 0.096 | -0.117 | 0.665 | -0.014 | 0.964 | 0.391 | 0.167 |
|  |  | *TTP dia* | *base* | 0.033 | 0.911 | -0.103 | 0.703 | **-0.657** | **0.006** | -0.142 | 0.601 | -0.083 | 0.788 | **0.605** | **0.022** |
|  |  |  | *mid* | 0.080 | 0.786 | 0.087 | 0.748 | **-0.601** | **0.014** | 0.021 | 0.939 | 0.235 | 0.439 | **0.750** | **0.002** |
|  |  |  | *apex* | 0.146 | 0.618 | 0.117 | 0.666 | -0.449 | 0.081 | 0.258 | 0.335 | 0.260 | 0.391 | **0.742** | **0.002** |
|  | *Long-axis (Vz)* | *peak sys* | *base* | -0.248 | 0.392 | -0.188 | 0.485 | 0.135 | 0.617 | -0.244 | 0.362 | -0.060 | 0.845 | -0.081 | 0.782 |
|  |  |  | *mid* | -0.191 | 0.513 | -0.358 | 0.174 | -0.315 | 0.235 | -0.062 | 0.820 | 0.016 | 0.957 | 0.011 | 0.970 |
|  |  |  | *apex* | -0.121 | 0.681 | **-0.634** | **0.008** | **-0.779** | **<0.001** | -0.050 | 0.854 | 0.060 | 0.845 | 0.112 | 0.703 |
|  |  | *peak dia* | *base* | 0.029 | 0.923 | 0.131 | 0.629 | 0.312 | 0.240 | -0.194 | 0.471 | -0.176 | 0.566 | -0.349 | 0.221 |
|  |  |  | *mid* | -0.174 | 0.553 | 0.097 | 0.720 | 0.259 | 0.333 | -0.279 | 0.295 | -0.368 | 0.216 | -0.191 | 0.513 |
|  |  |  | *apex* | 0.213 | 0.464 | 0.336 | 0.204 | 0.371 | 0.158 | -0.344 | 0.192 | **-0.731** | **0.005** | 0.002 | 0.994 |
|  |  | *TTP sys* | *base* | -0.056 | 0.850 | -0.019 | 0.943 | -0.442 | 0.087 | -0.064 | 0.813 | 0.220 | 0.470 | **0.635** | **0.015** |
|  |  |  | *mid* | 0.138 | 0.637 | 0.054 | 0.841 | -0.436 | 0.091 | -0.061 | 0.822 | 0.173 | 0.573 | **0.625** | **0.017** |
|  |  |  | *apex* | 0.195 | 0.504 | 0.290 | 0.276 | -0.147 | 0.587 | -0.378 | 0.149 | 0.136 | 0.658 | **0.550** | **0.042** |
|  |  | *TTP dia* | *base* | 0.135 | 0.645 | 0.267 | 0.317 | -0.397 | 0.127 | -0.040 | 0.882 | -0.118 | 0.702 | **0.760** | **0.002** |
|  |  |  | *mid* | 0.159 | 0.586 | 0.185 | 0.493 | **-0.520** | **0.039** | -0.010 | 0.970 | 0.122 | 0.692 | **0.592** | **0.026** |
|  |  |  | *apex* | 0.234 | 0.421 | 0.162 | 0.548 | -0.432 | 0.095 | -0.209 | 0.437 | -0.377 | 0.204 | 0.452 | 0.105 |
|  | *Circumferential (Vφ)* | *peak sys* | *base* | 0.451 | 0.106 | 0.063 | 0.816 | -0.479 | 0.060 | 0.068 | 0.803 | 0.011 | 0.972 | 0.090 | 0.759 |
|  |  |  | *mid* | 0.530 | 0.051 | 0.280 | 0.294 | -0.362 | 0.169 | 0.047 | 0.863 | -0.033 | 0.915 | 0.305 | 0.288 |
|  |  |  | *apex* | 0.486 | 0.078 | 0.349 | 0.185 | -0.371 | 0.158 | 0.003 | 0.991 | 0.071 | 0.817 | 0.222 | 0.446 |
|  |  | *peak dia* | *base* | -0.640 | 0.014 | -0.405 | 0.120 | 0.276 | 0.300 | 0.297 | 0.264 | 0.198 | 0.517 | -0.503 | 0.067 |
|  |  |  | *mid* | -0.451 | 0.106 | -0.440 | 0.088 | 0.212 | 0.431 | 0.206 | 0.444 | 0.071 | 0.817 | **-0.653** | **0.011** |
|  |  |  | *apex* | -0.077 | 0.794 | -0.104 | 0.700 | 0.262 | 0.327 | 0.297 | 0.264 | 0.324 | 0.280 | -0.385 | 0.175 |
|  |  | *TTP sys* | *base* | -0.041 | 0.889 | 0.285 | 0.285 | -0.021 | 0.937 | 0.338 | 0.200 | -0.056 | 0.855 | 0.342 | 0.231 |
|  |  |  | *mid* | -0.121 | 0.680 | 0.064 | 0.814 | -0.033 | 0.904 | 0.290 | 0.276 | -0.101 | 0.744 | 0.347 | 0.224 |
|  |  |  | *apex* | 0.301 | 0.296 | 0.327 | 0.216 | -0.150 | 0.578 | 0.206 | 0.445 | 0.083 | 0.787 | 0.306 | 0.287 |
|  |  | *TTP dia* | *base* | -0.233 | 0.422 | 0.071 | 0.792 | -0.259 | 0.332 | **-0.594** | **0.015** | -0.234 | 0.441 | 0.260 | 0.370 |
|  |  |  | *mid* | 0.000 | 1.000 | -0.112 | 0.680 | **-0.523** | **0.038** | -0.315 | 0.234 | -0.185 | 0.546 | 0.493 | 0.073 |
|  |  |  | *apex* | 0.152 | 0.603 | -0.218 | 0.418 | **-0.642** | **0.007** | -0.346 | 0.189 | -0.067 | 0.829 | 0.413 | 0.142 |

*LV* left ventricle, *peak sys* peak systolic velocity, *peak dia* peak diastolic velocity, *RV* right ventricle, *TTP dia* diastolic time to peak, *TTP sys* systolic time to peak. Significant correlations are indicated in bold script.

**Tab. S5b:** Spearman correlations between radial, long-axis, and circumferential tissue phase mapping and native T1 mapping in the left ventricle.

|  |  |  |  | **Native T1 mapping** | | | | | | | | | | | |
| --- | --- | --- | --- | --- | --- | --- | --- | --- | --- | --- | --- | --- | --- | --- | --- |
|  |  |  |  | *LV global* | | *Septum* | | *LV lateral* | | *RVOT* | | *RV free wall* | | *RV inferior* | |
|  |  |  |  | *r* | *p* | *r* | *p* | *r* | *p* | *r* | *p* | *r* | *p* | *r* | *p* |
| **Tissue phase mapping – left ventricle** | *Radial (Vr)* | *peak sys* | *base* | -0.068 | 0.817 | 0.208 | 0.441 | 0.241 | 0.368 | 0.365 | 0.165 | 0.033 | 0.915 | 0.152 | 0.605 |
|  |  |  | *mid* | -0.468 | 0.091 | -0.128 | 0.637 | 0.374 | 0.154 | -0.200 | 0.458 | -0.242 | 0.426 | -0.341 | 0.233 |
|  |  |  | *apex* | -0.108 | 0.714 | -0.205 | 0.447 | 0.379 | 0.147 | -0.024 | 0.931 | -0.264 | 0.384 | -0.099 | 0.737 |
|  |  | *peak dia* | *base* | 0.160 | 0.584 | 0.010 | 0.970 | -0.253 | 0.345 | 0.109 | 0.688 | 0.060 | 0.845 | -0.064 | 0.829 |
|  |  |  | *mid* | 0.310 | 0.281 | 0.409 | 0.116 | 0.150 | 0.579 | -0.250 | 0.350 | 0.132 | 0.668 | -0.029 | 0.923 |
|  |  |  | *apex* | 0.002 | 0.994 | -0.018 | 0.948 | -0.382 | 0.144 | -0.206 | 0.444 | -0.214 | 0.482 | 0.152 | 0.605 |
|  |  | *TTP sys* | *base* | 0.530 | 0.051 | 0.322 | 0.224 | -0.239 | 0.373 | 0.175 | 0.518 | 0.067 | 0.829 | **0.733** | **0.003** |
|  |  |  | *mid* | 0.149 | 0.612 | 0.121 | 0.654 | **-0.499** | **0.049** | -0.092 | 0.735 | 0.028 | 0.929 | **0.648** | **0.012** |
|  |  |  | *apex* | -0.029 | 0.922 | 0.080 | 0.770 | -0.396 | 0.129 | 0.031 | 0.908 | 0.143 | 0.642 | **0.687** | **0.007** |
|  |  | *TTP dia* | *base* | 0.243 | 0.403 | 0.126 | 0.643 | **-0.526** | **0.036** | -0.118 | 0.663 | 0.022 | 0.943 | **0.662** | **0.010** |
|  |  |  | *mid* | 0.144 | 0.623 | 0.067 | 0.804 | **-0.545** | **0.029** | -0.197 | 0.465 | 0.017 | 0.957 | **0.695** | **0.006** |
|  |  |  | *apex* | 0.132 | 0.653 | 0.087 | 0.750 | **-0.534** | **0.033** | -0.130 | 0.632 | 0.000 | 1.000 | **0.640** | **0.014** |
|  | *Long-axis (Vz)* | *peak sys* | *base* | -0.459 | 0.098 | -0.246 | 0.359 | 0.347 | 0.188 | -0.171 | 0.528 | -0.121 | 0.694 | -0.169 | 0.563 |
|  |  |  | *mid* | -0.415 | 0.140 | -0.344 | 0.192 | 0.185 | 0.492 | 0.053 | 0.846 | -0.121 | 0.694 | -0.002 | 0.994 |
|  |  |  | *apex* | -0.182 | 0.533 | -0.341 | 0.196 | -0.021 | 0.940 | -0.018 | 0.948 | -0.143 | 0.642 | 0.235 | 0.418 |
|  |  | *peak dia* | *base* | -0.033 | 0.911 | 0.135 | 0.617 | 0.082 | 0.762 | -0.218 | 0.418 | -0.319 | 0.289 | -0.051 | 0.864 |
|  |  |  | *mid* | 0.138 | 0.637 | -0.059 | 0.829 | -0.079 | 0.770 | -0.432 | 0.094 | -0.418 | 0.156 | -0.213 | 0.464 |
|  |  |  | *apex* | -0.007 | 0.982 | 0.112 | 0.680 | -0.206 | 0.444 | 0.112 | 0.680 | 0.247 | 0.415 | -0.213 | 0.464 |
|  |  | *TTP sys* | *base* | 0.034 | 0.908 | 0.025 | 0.927 | -0.409 | 0.116 | -0.088 | 0.747 | 0.043 | 0.890 | 0.528 | 0.052 |
|  |  |  | *mid* | 0.215 | 0.461 | 0.197 | 0.465 | -0.486 | 0.056 | 0.017 | 0.951 | 0.119 | 0.699 | **0.719** | **0.004** |
|  |  |  | *apex* | 0.181 | 0.537 | 0.108 | 0.690 | -0.408 | 0.117 | -0.280 | 0.293 | -0.107 | 0.728 | 0.441 | 0.114 |
|  |  | *TTP dia* | *base* | 0.148 | 0.613 | 0.128 | 0.636 | **-0.524** | **0.037** | -0.092 | 0.735 | 0.014 | 0.964 | **0.650** | **0.012** |
|  |  |  | *mid* | 0.131 | 0.656 | 0.165 | 0.542 | -0.448 | 0.082 | -0.053 | 0.844 | 0.019 | 0.950 | **0.608** | **0.021** |
|  |  |  | *apex* | 0.183 | 0.531 | -0.098 | 0.717 | **-0.534** | **0.033** | -0.177 | 0.511 | -0.262 | 0.387 | 0.263 | 0.363 |
|  | *Circumferential (Vφ)* | *peak sys* | *base* | 0.429 | 0.126 | 0.084 | 0.757 | -0.212 | 0.431 | -0.021 | 0.940 | -0.247 | 0.415 | 0.349 | 0.221 |
|  |  |  | *mid* | 0.490 | 0.075 | 0.037 | 0.892 | -0.362 | 0.169 | 0.191 | 0.478 | 0.016 | 0.957 | 0.451 | 0.106 |
|  |  |  | *apex* | 0.473 | 0.088 | 0.171 | 0.527 | -0.453 | 0.078 | 0.009 | 0.974 | -0.016 | 0.957 | 0.393 | 0.164 |
|  |  | *peak dia* | *base* | -0.495 | 0.072 | **-0.667** | **0.005** | -0.003 | 0.991 | -0.018 | 0.948 | -0.093 | 0.762 | **-0.547** | **0.043** |
|  |  |  | *mid* | **-0.653** | **0.011** | -0.386 | 0.140 | 0.171 | 0.528 | -0.062 | 0.820 | -0.049 | 0.873 | **-0.662** | **0.010** |
|  |  |  | *apex* | -0.376 | 0.185 | -0.063 | 0.816 | 0.344 | 0.192 | -0.071 | 0.795 | -0.253 | 0.405 | -0.380 | 0.180 |
|  |  | *TTP sys* | *base* | 0.020 | 0.947 | 0.374 | 0.153 | -0.134 | 0.620 | 0.070 | 0.798 | -0.080 | 0.795 | 0.496 | 0.072 |
|  |  |  | *mid* | -0.073 | 0.803 | 0.129 | 0.635 | -0.138 | 0.610 | 0.071 | 0.793 | -0.111 | 0.719 | **0.570** | **0.033** |
|  |  |  | *apex* | -0.262 | 0.366 | 0.130 | 0.632 | -0.221 | 0.411 | 0.034 | 0.902 | -0.131 | 0.670 | **0.691** | **0.006** |
|  |  | *TTP dia* | *base* | 0.143 | 0.627 | 0.238 | 0.376 | -0.236 | 0.379 | -0.401 | 0.124 | -0.273 | 0.367 | 0.209 | 0.473 |
|  |  |  | *mid* | 0.002 | 0.994 | 0.137 | 0.612 | -0.328 | 0.215 | -0.410 | 0.114 | -0.204 | 0.503 | 0.490 | 0.076 |
|  |  |  | *apex* | 0.233 | 0.423 | -0.082 | 0.763 | **-0.542** | **0.030** | -0.343 | 0.193 | -0.088 | 0.774 | 0.435 | 0.120 |

*LV* left ventricle, *peak sys* peak systolic velocity, *peak dia* peak diastolic velocity, *RV* right ventricle, *TTP dia* diastolic time to peak, *TTP sys* systolic time to peak. Significant correlations are indicated in bold script.

**Tab. S6:** Spearman correlations between tissue phase parameters and age in patients with rTOF and healthy volunteers (‘controls’).

|  |  |  |  | **Age** | | | | | | | | | |
| --- | --- | --- | --- | --- | --- | --- | --- | --- | --- | --- | --- | --- | --- |
|  |  |  |  |  | *TOF* | | *controls* | |  | *TOF* | | *controls* | |
|  |  |  |  |  | *r* | *p* | *r* | *p* |  | *r* | *p* | *r* | *p* |
| **Tissue phase mapping** | *Radial (Vr)* | *peak sys* | *base* | *Right ventricle* | -0.229 | 0.394 | -0.112 | 0.594 | *Left ventricle* | 0.176 | 0.516 | -0.124 | 0.553 |
|  |  |  | *mid* |  | -0.009 | 0.974 | 0.310 | 0.132 |  | 0.273 | 0.307 | 0.106 | 0.616 |
|  |  |  | *apex* |  | 0.171 | 0.526 | 0.119 | 0.570 |  | -0.100 | 0.712 | 0.055 | 0.794 |
|  |  | *peak dia* | *base* |  | 0.142 | 0.601 | 0.284 | 0.169 |  | -0.243 | 0.364 | 0.139 | 0.506 |
|  |  |  | *mid* |  | 0.316 | 0.234 | 0.092 | 0.662 |  | 0.251 | 0.349 | 0.159 | 0.448 |
|  |  |  | *apex* |  | -0.106 | 0.695 | 0.158 | 0.451 |  | 0.469 | 0.067 | 0.532 | **0.006** |
|  |  | *TTP sys* | *base* |  | 0.187 | 0.488 | -0.086 | 0.683 |  | 0.128 | 0.637 | -0.063 | 0.763 |
|  |  |  | *mid* |  | 0.098 | 0.719 | -0.231 | 0.267 |  | 0.209 | 0.436 | -0.163 | 0.436 |
|  |  |  | *apex* |  | 0.071 | 0.792 | 0.288 | 0.163 |  | 0.176 | 0.514 | 0.363 | 0.074 |
|  |  | *TTP dia* | *base* |  | 0.298 | 0.262 | -0.265 | 0.201 |  | 0.393 | 0.132 | -0.194 | 0.354 |
|  |  |  | *mid* |  | 0.238 | 0.375 | -0.034 | 0.872 |  | 0.349 | 0.185 | 0.022 | 0.917 |
|  |  |  | *apex* |  | 0.247 | 0.356 | 0.052 | 0.806 |  | 0.311 | 0.241 | 0.144 | 0.492 |
|  | *Long-axis (Vz)* | *peak sys* | *base* |  | -0.167 | 0.537 | 0.133 | 0.527 |  | 0.010 | 0.970 | -0.112 | 0.593 |
|  |  |  | *mid* |  | 0.106 | 0.695 | -0.056 | 0.791 |  | 0.004 | 0.987 | 0.062 | 0.767 |
|  |  |  | *apex* |  | 0.347 | 0.188 | 0.082 | 0.697 |  | 0.202 | 0.453 | 0.215 | 0.302 |
|  |  | *peak dia* | *base* |  | 0.432 | 0.095 | -0.056 | 0.789 |  | 0.504 | **0.046** | -0.147 | 0.485 |
|  |  |  | *mid* |  | 0.299 | 0.260 | 0.043 | 0.838 |  | 0.183 | 0.498 | 0.135 | 0.520 |
|  |  |  | *apex* |  | 0.186 | 0.491 | -0.020 | 0.924 |  | -0.355 | 0.177 | -0.025 | 0.905 |
|  |  | *TTP sys* | *base* |  | -0.028 | 0.917 | 0.099 | 0.637 |  | 0.201 | 0.456 | -0.015 | 0.942 |
|  |  |  | *mid* |  | -0.073 | 0.788 | 0.077 | 0.715 |  | 0.278 | 0.297 | 0.026 | 0.903 |
|  |  |  | *apex* |  | 0.018 | 0.946 | 0.038 | 0.857 |  | 0.202 | 0.454 | 0.393 | 0.052 |
|  |  | *TTP dia* | *base* |  | 0.408 | 0.117 | -0.185 | 0.376 |  | 0.351 | 0.183 | -0.039 | 0.854 |
|  |  |  | *mid* |  | 0.274 | 0.305 | 0.161 | 0.442 |  | 0.400 | 0.125 | 0.071 | 0.735 |
|  |  |  | *apex* |  | 0.395 | 0.130 | -0.029 | 0.889 |  | 0.139 | 0.609 | 0.086 | 0.682 |
|  | *Circumferential (Vφ)* | *peak sys* | *base* |  | 0.044 | 0.871 | 0.044 | 0.834 |  | 0.510 | **0.043** | 0.293 | 0.156 |
|  |  |  | *mid* |  | 0.111 | 0.683 | 0.412 | **0.041** |  | 0.214 | 0.426 | 0.243 | 0.241 |
|  |  |  | *apex* |  | -0.021 | 0.939 | 0.459 | **0.021** |  | 0.249 | 0.352 | 0.121 | 0.565 |
|  |  | *peak dia* | *base* |  | 0.063 | 0.815 | -0.037 | 0.860 |  | -0.063 | 0.815 | 0.137 | 0.514 |
|  |  |  | *mid* |  | 0.099 | 0.716 | -0.133 | 0.525 |  | 0.009 | 0.974 | -0.128 | 0.542 |
|  |  |  | *apex* |  | -0.087 | 0.749 | -0.044 | 0.834 |  | -0.072 | 0.790 | 0.105 | 0.617 |
|  |  | *TTP sys* | *base* |  | 0.350 | 0.184 | 0.034 | 0.873 |  | 0.417 | 0.108 | 0.054 | 0.796 |
|  |  |  | *mid* |  | 0.139 | 0.609 | 0.236 | 0.255 |  | 0.312 | 0.239 | 0.148 | 0.481 |
|  |  |  | *apex* |  | -0.360 | 0.171 | -0.104 | 0.622 |  | 0.621 | **0.010** | -0.180 | 0.389 |
|  |  | *TTP dia* | *base* |  | 0.576 | **0.020** | 0.181 | 0.387 |  | 0.363 | 0.167 | 0.107 | 0.612 |
|  |  |  | *mid* |  | 0.620 | **0.010** | -0.042 | 0.841 |  | 0.587 | **0.017** | 0.323 | 0.115 |
|  |  |  | *apex* |  | 0.343 | 0.194 | 0.164 | 0.434 |  | 0.394 | 0.131 | 0.014 | 0.949 |

*Peak sys* peak systolic velocity, *peak dia* peak diastolic velocity, *RV* right ventricle, *TTP dia* diastolic time to peak, *TTP sys* systolic time to peak. Significant correlations are indicated in bold script.
